# Supplementary material for: HEalth professionals Responding to MEn for Safety (HERMES): Mixed methods evaluation of a pilot sexual health intervention for gay, bisexual and other men who have sex with men experiencing domestic violence and abuse
Source: PLoS One. 2025 Jan 8;20(1):e0312807. doi: 10.1371/journal.pone.0312807 (PMC11709232; doi:10.1371/journal.pone.0312807)
Supplement: S1 Fig — (PDF) [file pone.0312807.s001.pdf]

Hermes model for Identification and Referral for MSM DV – AFC

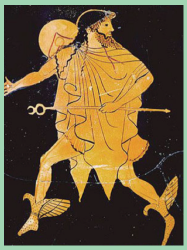

1 Patient asks for help with **domestic violence (DV)**
(DIRECT REFERRAL TO HA)

2 Prompting conditions/ symptoms/problems

- Anger problem (him/partner)
- Relationship conflict/asking for couple counselling
- Asking if partner has visited clinic
- Fear partner will out them
- Unexplained injuries (esp. groin/face)
- Depression and/or suicidality
- Drugs or alcohol problems
- Penile dysfunction
- Refusal to use condoms
- Repeat STIs
- Other sexual health problems

3 Follow up questions

- How do you feel about your relationship?
- Have there been any problems lately in your relationship with your partner?
- Have there been any frightening arguments?
- Has any physical violence been used? (e.g. hitting, slapping, kicking, shoving)
- Did you know that there are people who can help you (and your partner/ children) if there is violence in your relationship, for men as well as women? I can tell you about these

4 Indirect indicators of DV in the absence of disclosure

- Patient fear of partner or of consequences of partner's decisions about relationship
- Fear of what partner will say or do if they make certain decisions
- Responding to questions by asking for help even without explicit disclosure

5 Documentation on AFC Proforma

NEVER LEAVE DV BOX BLANK!

History of DV:
Yes/No/Declines to answer

OR tick

"Not asked as no symptoms"

6 Refer to HA if …

- DV disclosed
- Any other two indicators of DV from Box 4 present

7 Referral Agency

GALOP (www.galop.org.uk)

Helpline: 020 7704 2040
10 am – 4pm Mon to Fri

Client may also report themselves online:
www/gallop.org.uk/online-report-form
